# Supplementary material for: Measuring correlation and entanglement between molecular orbitals on a trapped-ion quantum computer
Source: Sci Rep. 2025 Aug 4;15:28409. doi: 10.1038/s41598-025-04365-x (PMC12322021; doi:10.1038/s41598-025-04365-x)
Supplement: Supplementary file 1 — Supplementary Information. [file 41598_2025_4365_MOESM1_ESM.pdf]

Supplementary Information for "Measuring Correlation and Entanglement between Molecular Orbitals on a Trapped-Ion Quantum Computer"

Gabriel Greene-Diniz,[1] Chris N. Self,[2] Michal Krompiec,[1] Luuk Coopmans,[2]
Marcello Benedetti,[2] David Muñoz Ramo,[1] and Matthias Rosenkranz[2]

[1] *Quantinuum, Terrington House, 13-15 Hills Road, Cambridge CB2 1NL, United Kingdom*
[2] *Quantinuum, Partnership House, Carlisle Place, London SW1P 1BX, United Kingdom*

Appendix A: Fermionic operators for the ORDMs

Here we give the 16 elements of the 1-ORDM in terms of the fermionic operators of the molecular orbitals:

$$\mathcal{O}(i)_1 = 1 - \hat{n}_{i,\uparrow} - \hat{n}_{i,\downarrow} + \hat{n}_{i,\uparrow}\hat{n}_{i,\downarrow} \tag{A1}$$
$$\mathcal{O}(i)_2 = \hat{f}_{i,\downarrow} - \hat{n}_{i,\uparrow}\hat{f}_{i,\downarrow} \tag{A2}$$
$$\mathcal{O}(i)_3 = \hat{f}_{i,\uparrow} - \hat{n}_{i,\downarrow}\hat{f}_{i,\uparrow} \tag{A3}$$
$$\mathcal{O}(i)_4 = \hat{f}_{i,\downarrow}\hat{f}_{i,\uparrow} \tag{A4}$$
$$\mathcal{O}(i)_5 = \hat{f}^\dagger_{i,\downarrow} - \hat{n}_{i,\uparrow}\hat{f}^\dagger_{i,\downarrow} \tag{A5}$$
$$\mathcal{O}(i)_6 = \hat{n}_{i,\downarrow} - \hat{n}_{i,\uparrow}\hat{n}_{i,\downarrow} \tag{A6}$$
$$\mathcal{O}(i)_7 = \hat{f}^\dagger_{i,\downarrow}\hat{f}_{i,\uparrow} \tag{A7}$$
$$\mathcal{O}(i)_8 = \text{-}\hat{n}_{i,\downarrow}\hat{f}_{i,\uparrow} \tag{A8}$$
$$\mathcal{O}(i)_9 = \hat{f}^\dagger_{i,\uparrow} - \hat{n}_{i,\downarrow}\hat{f}^\dagger_{i,\uparrow} \tag{A9}$$
$$\mathcal{O}(i)_{10} = \hat{f}_{i,\downarrow}\hat{f}^\dagger_{i,\uparrow} \tag{A10}$$
$$\mathcal{O}(i)_{11} = \hat{n}_{i,\uparrow} - \hat{n}_{i,\uparrow}\hat{n}_{i,\downarrow} \tag{A11}$$
$$\mathcal{O}(i)_{12} = \hat{n}_{i,\uparrow}\hat{f}_{i,\downarrow} \tag{A12}$$
$$\mathcal{O}(i)_{13} = \hat{f}^\dagger_{i,\downarrow}\hat{f}^\dagger_{i,\uparrow} \tag{A13}$$
$$\mathcal{O}(i)_{14} = \text{-}\hat{n}_{i,\downarrow}\hat{f}^\dagger_{i,\uparrow} \tag{A14}$$
$$\mathcal{O}(i)_{15} = \hat{n}_{i,\uparrow}\hat{f}^\dagger_{i,\downarrow} \tag{A15}$$
$$\mathcal{O}(i)_{16} = \hat{n}_{i,\uparrow}\hat{n}_{i,\downarrow}, \tag{A16}$$

where $\hat{f}_{i,\sigma}$ and $\hat{n}_{i,\sigma}$ are the annihilation operator and number operator for spin orbital σ of molecular orbital i.

Appendix B: Qubit operators for the ORDMs

We map the up and down modes of fermionic orbital i to a pair of qubits $(2i, 2i+1)$ by JW transformation, using the convention

$$\hat{f}_{i,\uparrow} = \frac{1}{2}\Big(X_{2i} + iY_{2i}\Big)\prod_{k>2i} Z_k \tag{B1}$$
$$\hat{f}_{i,\downarrow} = \frac{1}{2}\Big(X_{2i+1} + iY_{2i+1}\Big)\prod_{k>2i+1} Z_k. \tag{B2}$$

The qubit operators for the diagonal elements of the 1-ORDM matrix are given by:

$$\mathcal{O}(i)_1 = \frac{1}{4}\Big(\mathbb{I} + Z_{2i} + Z_{2i+1} + Z_{2i}Z_{2i+1}\Big) \tag{B3}$$

$$\mathcal{O}(i)_6 = \frac{1}{4}\Big(\mathbb{I} + Z_{2i} - Z_{2i+1} - Z_{2i}Z_{2i+1}\Big) \tag{B4}$$

$$\mathcal{O}(i)_{11} = \frac{1}{4}\Big(\mathbb{I} - Z_{2i} + Z_{2i+1} - Z_{2i}Z_{2i+1}\Big) \tag{B5}$$

$$\mathcal{O}(i)_{16} = \frac{1}{4}\Big(\mathbb{I} - Z_{2i} - Z_{2i+1} + Z_{2i}Z_{2i+1}\Big). \tag{B6}$$

Instead of giving the full expressions for the 2-ORDM qubit operators, we list the remaining $\mathcal{O}(i)_k$ needed to construct each of them

$$\mathcal{O}(i)_2 = \frac{1}{4}\Big(X_{2i+1} + iY_{2i+1} + Z_{2i}X_{2i+1} + iZ_{2i}Y_{2i+1}\Big) \prod_{k>2i+1} Z_k \tag{B7}$$

$$\mathcal{O}(i)_3 = \frac{1}{4}\Big(X_{2i} + iY_{2i} + X_{2i}Z_{2i+1} + iY_{2i}Z_{2i+1}\Big) \prod_{k>2i+1} Z_k \tag{B8}$$

$$\mathcal{O}(i)_4 = \frac{1}{4}\Big((1-i)\,Y_{2i}Y_{2i+1} - (1-i)\,X_{2i}X_{2i+1}\Big) \tag{B9}$$

$$\mathcal{O}(i)_5 = \frac{1}{4}\Big(X_{2i+1} - iY_{2i+1} + Z_{2i}X_{2i+1} - iZ_{2i}Y_{2i+1}\Big) \prod_{k>2i+1} Z_k \tag{B10}$$

$$\mathcal{O}(i)_7 = \frac{1}{4}\Big(iY_{2i}X_{2i+1} - iX_{2i}Y_{2i+1} + X_{2i}X_{2i+1} + Y_{2i}Y_{2i+1}\Big) \tag{B11}$$

$$\mathcal{O}(i)_8 = \frac{1}{4}\Big(X_{2i} + iY_{2i} - X_{2i}Z_{2i+1} - iY_{2i}Z_{2i+1}\Big) \prod_{k>2i+1} Z_k \tag{B12}$$

$$\mathcal{O}(i)_9 = \frac{1}{4}\Big(X_{2i} - iY_{2i} + X_{2i}Z_{2i+1} - iY_{2i}Z_{2i+1}\Big) \prod_{k>2i+1} Z_k \tag{B13}$$

$$\mathcal{O}(i)_{10} = \frac{1}{4}\Big(iY_{2i}X_{2i+1} - iX_{2i}Y_{2i+1} - X_{2i}X_{2i+1} - Y_{2i}Y_{2i+1}\Big) \tag{B14}$$

$$\mathcal{O}(i)_{12} = \frac{1}{4}\Big(X_{2i+1} + iY_{2i+1} - Z_{2i}X_{2i+1} - iZ_{2i}Y_{2i+1}\Big) \prod_{k>2i+1} Z_k \tag{B15}$$

$$\mathcal{O}(i)_{13} = \frac{1}{4}\Big((1-i)\,X_{2i}X_{2i+1} - (1+i)\,Y_{2i}Y_{2i+1}\Big) \tag{B16}$$

$$\mathcal{O}(i)_{14} = \frac{1}{4}\Big(X_{2i} - iY_{2i} - X_{2i}Z_{2i+1} + iY_{2i}Z_{2i+1}\Big) \prod_{k>2i+1} Z_k \tag{B17}$$

$$\mathcal{O}(i)_{15} = \frac{1}{4}\Big(X_{2i+1} - iY_{2i+1} - Z_{2i}X_{2i+1} + iZ_{2i}Y_{2i+1}\Big) \prod_{k>2i+1} Z_k. \tag{B18}$$

Appendix C: Chemical State Circuits

The optimized VQE circuits used to generate the ground state of image 1 of the NEB path in the singlet and triplet configurations are shown in Fig. S1. The statevectors of these circuits are equivalent to the second row, second column cell of (a) Table I and (b) Table II of the main text.

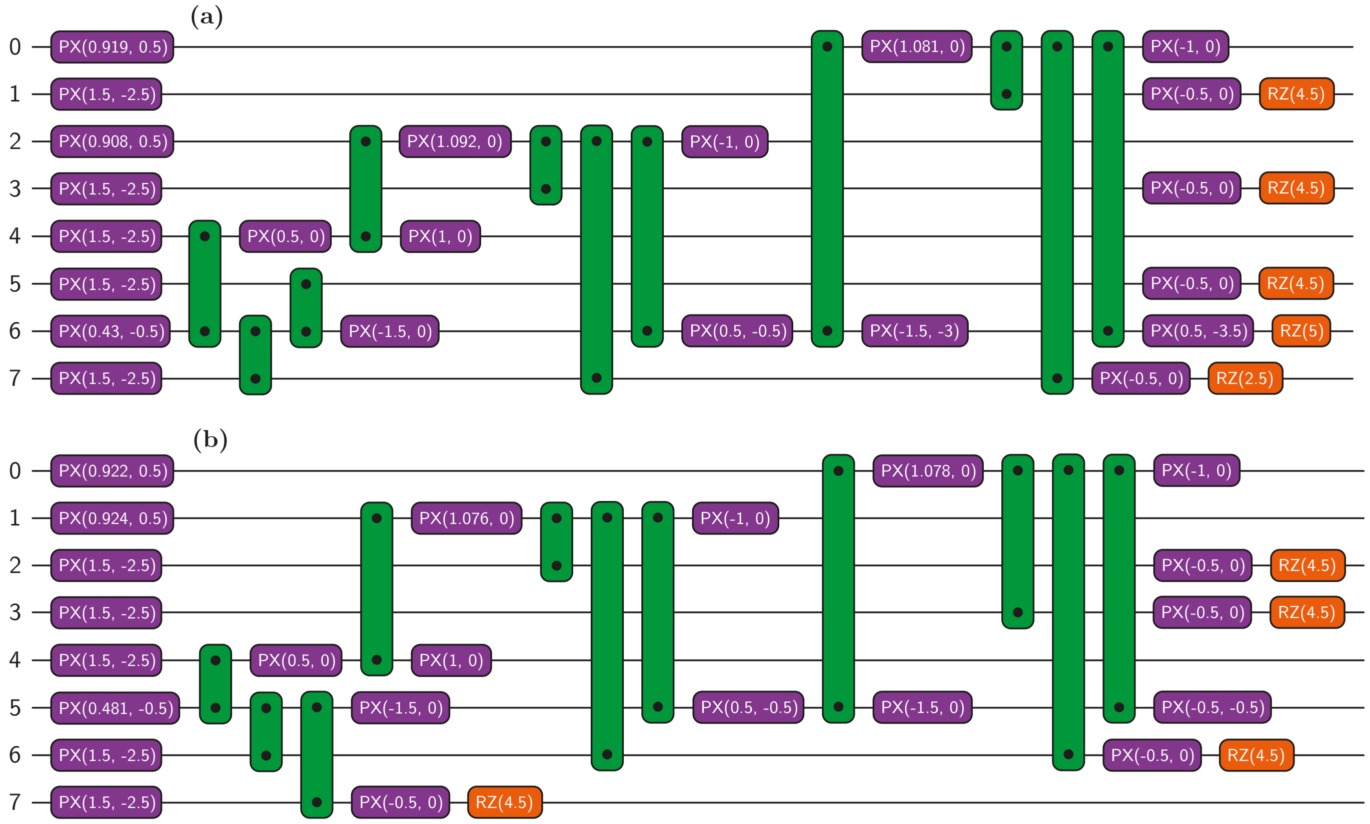

FIG. S1. Circuits to represent (a) the $\langle S^2 \rangle = 0$ wavefunction and (b) the $\langle S^2 \rangle = 2$ wavefunction, both corresponding to the 8 qubit O_2 p-orbital projected AVAS basis. Rotation angles (rounded to three decimal places) represent image 1 of the NEB path. The circuits are shown compiled to the H1-1 gate set [1]. The circuits contain single qubit rotations about the Z axes of the Bloch sphere (RZ), a more general single qubit rotation called phased-X that is equivalent to $\mathrm{PX}(\theta, \phi) = R_Z(-\phi) R_X(\theta) R_Z(\phi)$ and a maximally entangling two-qubit rotation gate $e^{-i\frac{\pi}{4} Z \otimes Z}$. All rotation angles shown on the circuit are given in units of π.

Appendix D: Density correction to the hard-threshold for singular value

To reduce noise in our measured ORDMs we hard-threshold the singular values of the measured matrices. Due to the reduced number of elements in the matrices we measure we find we need to adjust the optimal threshold reported in [2].

In [2] the authors consider $d \times d$ matrices of the form $Y = X + \sigma Z$ where X is the matrix whose singular values we want to find and Z is a noise matrix whose elements are independent, identically distributed random variables of mean 0. They show that asymptotically the optimal hard-threshold for removing the small singular values of Y caused by noise is $(4/\sqrt{3})\,\sqrt{d}\,\sigma$.

In characterizing the fermionic ORDM matrices we only measure m elements of the matrix, so that $d^2 - m$ entries of Y are zero. To compensate for this we find we should reduce the hard-threshold to $(4/\sqrt{3})\,\sqrt{dR}\,\sigma$, where $R = m/d^2$ is the density of non-zero elements in Y. We numerically observe that this reduced hard-threshold is appropriate by plotting the distribution of singular values for 1000 randomly generated matrices at different densities, R, in Fig. S2. We see that the "bulk-edge" of the noisy singular values decays with $\sqrt{R}$. Hence as the density of non-zero values in the matrix falls we should decrease the noise threshold too.

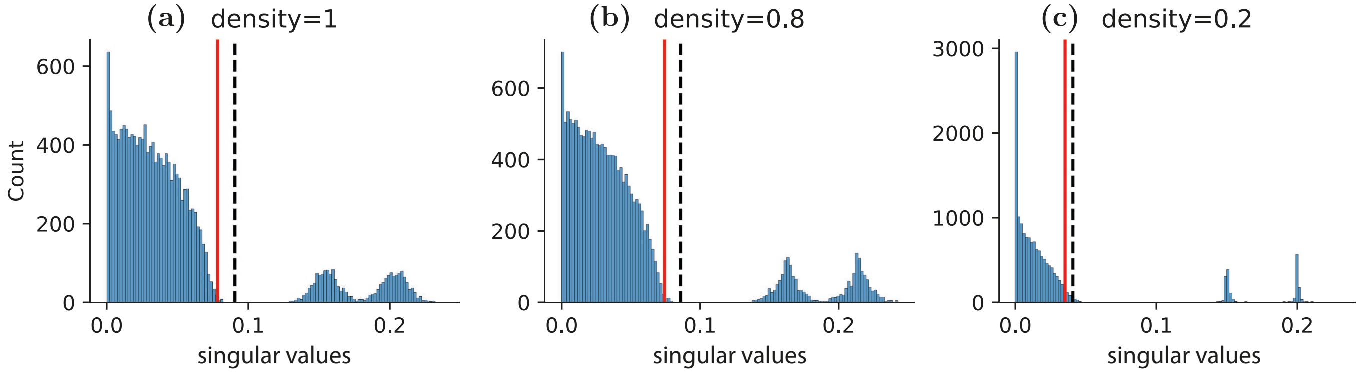

FIG. S2. Numerical investigation of the scaling of the "bulk-edge" of the singular values with the density of non-zero elements in a noisy matrix. For each density, R, we sample 1000 16×16 matrices of the form $Y = X + \sigma Z$ where X has all zero elements apart from $X_{1,1} = 0.15$ and $X_{2,2} = 0.2$ and $\sigma = 1/\sqrt{10000}$. The noise matrix Z is constructed by randomly selecting $m = \lfloor R \times 16^2 \rfloor$ elements and setting each of those elements to values sampled from a Normal distribution with mean 0 and variance 1, all other entries of Z are zero. Histograms for the singular values are shown at densities (a) $R = 1$, (b) $R = 0.8$ and (c) $R = 0.2$. On each plot the red vertical line indicates an apparent "bulk-edge" of the noisy singular values at $2\sqrt{dR}\,\sigma$ and the black dashed line is drawn at $(4/\sqrt{3})\sqrt{dR}\,\sigma$.

SUPPLEMENTARY REFERENCES

[1] Quantinuum H1-1, `https://www.quantinuum.com/`. H1-1 operates with 20 qubits each implemented through the $S_{1/2}$ hyperfine clock states of $^{171}Yb^{+}$. Ions are shuttled between 5 interaction zones in a linear trap design allowing for all-to-all connectivity. All circuits compiled, executed, and measured between June 2024 and August 2024, during which time the average hardware parameters were as follows; one-qubit gate infidelity: 2.1×10^{-5}, two-qubit gate infidelity: 8.8×10^{-4}, state preparation and measurement error: 2.5×10^{-3}.

[2] M. Gavish and D. L. Donoho, The optimal hard threshold for singular values is $4/\sqrt{3}$, IEEE Transactions on Information Theory **60**, 5040 (2014).
